# Examining associations between smartphone use, smartphone addiction, and mental health outcomes: A cross-sectional study of college students

**DOI:** 10.34172/hpp.2021.06

**Published:** 2021-02-07

**Authors:** Namyun Kil, Junhyoung Kim, Justin T. McDaniel, Jun Kim, Kari Kensinger

**Affiliations:** ^1^Department of Recreation Management and Therapeutic Recreation, University of Wisconsin-La Crosse, La Crosse, WI 54601, USA; ^2^Department of Health & Wellness Design, School of Public Health, Indiana University, Bloomington, IN 47405, USA; ^3^School of Human Sciences, Southern Illinois University, Carbondale, IL 62901, USA; ^4^Therapeutic Recreation of Nebraska, Omaha, NE 68127, USA

**Keywords:** Anxiety, Depression, Emotional distress, Satisfaction with life, Smartphone use, Smartphone addiction, Stress

## Abstract

**Background:** Prior studies have indicated the complex relationships of smartphone use and smartphone addiction with mental health and life satisfaction. The purpose of this study was to investigate the structural relationships among smartphone use, smartphone addiction, mental health problems (e.g., depression, anxiety, stress [DAS] and satisfaction with life [SWL]).

**Methods:** Cross-sectional data were collected by convenience sampling via an online survey of undergraduate students at a Midwestern university in the United States. The sample size of601 collected from undergraduate students that owned a smartphone and completed responses to the variables was utilized in this study. We assessed the hypothesized variables, including smartphone use, smartphone addiction, and mental health outcomes variables on a Likert-type scale. Structural analysis was used to examine the relationships.

**Results:** Results suggested that smartphone use had a significant negative association with DAS symptoms (β = -.31, t = -3.81, P < .001) and was positively associated with SWL (β =.25, t = 3.41, P < .001). However, smartphone use had a significant positive relationship with smartphone addiction (β = .48, t = 5.51, P < .001). Smartphone addiction was positively related to DAS (β = .44, t = 6.33, P < .001), but it was not related to SWL (β = -.08, t = -1.26, P > .05).

**Conclusion:** This study enhances our understanding of the associations between smartphone use and the health and well-being of undergraduate students. Implications for supporting their psychological health are discussed.

## Introduction

Rapid advancements in technology have led college students to spend considerable time using smartphones. According to Statista^1^, the number of college students who use smartphones in the United States (U.S.) is projected to reach 272.6 million in 2020 and 285.3 million by 2023, indicating that smartphones have become an inseparable part of their lives. Researchers have reported that college students use their smartphone for entertainment, stress reduction, education, games, navigation services, and social networking.^2,3^

Due to the increasing number of smartphone users and their needs, public health researchers have undertaken investigations into the effects of smartphone use on health. Some researchers suggest that smartphones help users form and develop social and global connections with others, gain knowledge and information, and entertain themselves through a variety of applications.^4,5^ On the other hand, the majority of previous studies present various negative physical and psychological consequences of excessive smartphone use including sleep deprivation, anxiety, stress, low self-esteem, and depression.^3,6-8^ For example, adolescents who actively engage in social media and smartphone use are more likely to experience high rates of hopelessness, suicidal ideation, and suicidal attempts.^9^ In addition, people who exhibit problematic smartphone use tend to be involved in risky situations (e.g., texting while driving) and use them in inappropriate social and educational settings (e.g., while in meetings, social meals or classrooms).^2,10-12^

In particular, college students are among the most vulnerable groups in the U.S. for developing addictive behaviors due to excessive smartphone use.^13^ There is evidence to suggest that college students are likely to engage in problematic smartphone use and, as a result, to experience negative physical and mental health outcomes, such as a lack of physical activity, low self-esteem, and decreased social engagement.^14,15^ In addition, previous studies have provided evidence that smartphone addiction is associated with emotional distress (e.g., high levels of anxiety and depression) among college students.^16-18^

Accumulating evidence shows contradictory findings on the relationship between the amount of smartphone use and psychological problems. These mixed results indicate the need for further research to investigate how smartphone use and addiction affect the mental health and life satisfaction of college students. For example, Samaha and Hawi^19^ showed that there was a negative correlation between smartphone addiction and satisfaction with life (SWL) among 300 university students. On the other hand, smartphone addiction was not a significant predictor of life satisfaction among Chinese college students.^20^ To help clarify this discrepancy in the literature, the present study is an investigation of the structural relationships among smartphone use, smartphone addiction, mental health problems (e.g., depression, anxiety, stress [DAS]), and SWL among college students.

### Associations between smartphone use, smartphone addiction, and mental health outcomes

Addiction is characterized by abnormally high reliance on a specific substance or activity causing repeated engagement in behaviors that have negative outcomes.^21^ Roberts et al^13^ proposed that smartphone activities are significantly associated with addiction due to users’ tendencies toward excessive usage. For example, Cha and Seo^22^ determined that the duration of daily smartphone and social networking use was related to smartphone addiction among Korean middle school students. To the extent that smartphones provide instant gratification through access to information, social interaction, and entertainment, smartphone users may become conditioned to repeatedly check their smartphones and, ultimately, become addicted.^22^

Smartphone usage has also been shown to be positively associated with “technostress,” which Lee et al^3^ describe as a “modern disease of adaptation caused by an inability to cope with new computer technologies in a healthy manner(p. 373)^.^”^3^ Prior studies support the idea that smartphone addiction is negatively associated with mental health among college students. Lepp, et al^23^ found that amount of smartphone usage, measured in minutes per day, was positively associated with distress among undergraduate college students. Demirci et al^16^ reported a positive association between smartphone addiction and depression among college students in Turkey. In a similar vein, Volungis et al^18^ found that smartphone addiction served as a significant predictor of emotional distress (e.g., depression, anxiety) among college students. Similarly, Vannucci et al^17^ found that greater use of social media was associated with increased likelihood of anxiety symptoms in individuals aged 18 to 22. Thus, smartphone addiction has been associated with a wide range of mental health problems among youth, including DAS.

**Figure 1:**
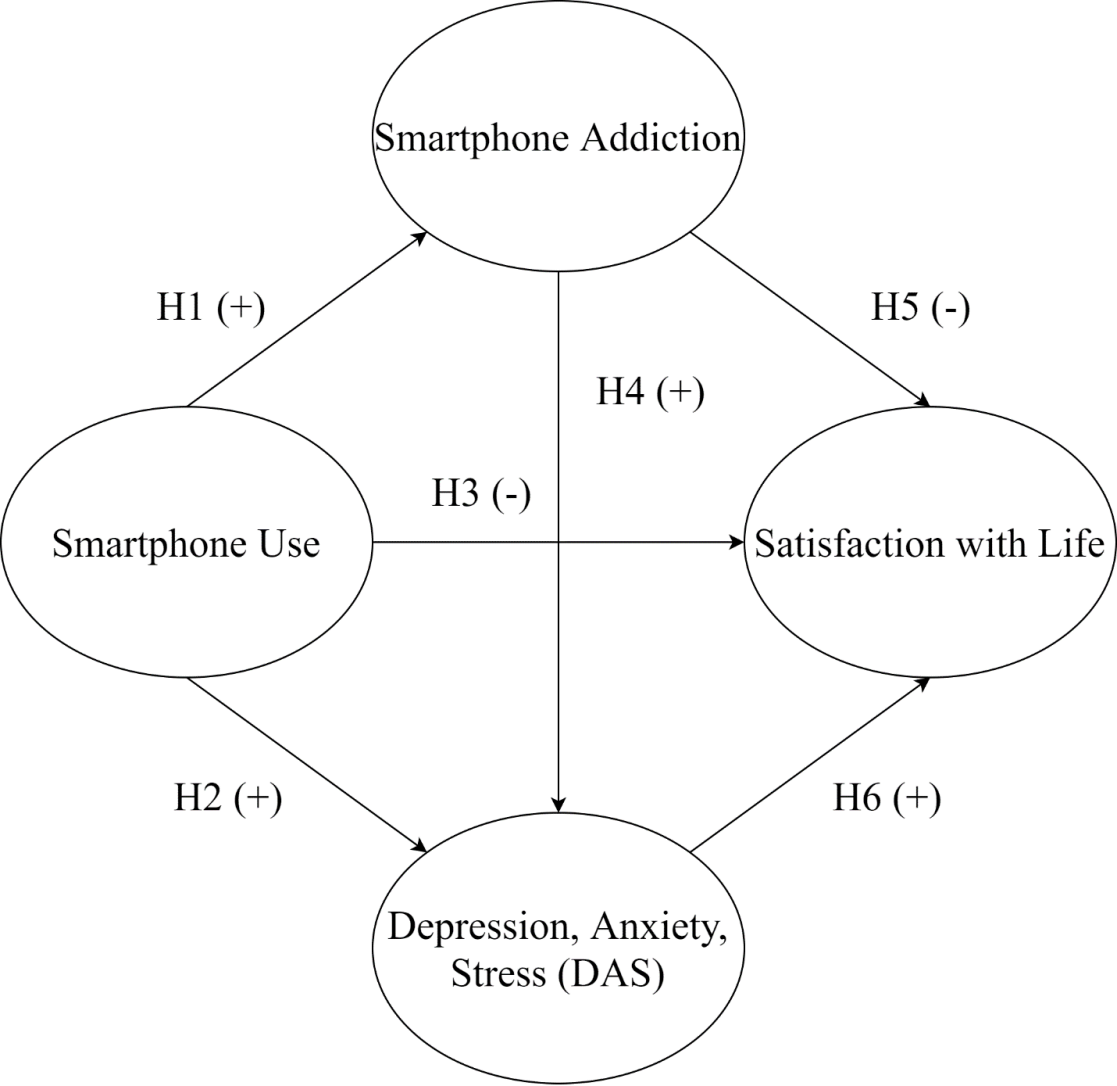
A proposed structural model with hypotheses.

A few studies on the relationship between smartphone use and SWL have presented mixed findings. For example, Lepp et al^7^ showed that college students who are smartphone users tend to experience high levels of anxiety and low-grade point averages that may result in diminished SWL. Also, Samaha and Hawi^19^ found that excessive smartphone use has a negative correlation with life satisfaction among college students. However, Yang et al^20^ did not find any negative or positive association between smartphone addiction and life satisfaction among college students in China. Thus, the relationship between smartphone addiction and life satisfaction is unclear and needs to be further examined. In addition, Kumar et al^24^ showed that as scores on DAS increased, scores on life satisfaction decreased among university students in Pakistan. However, while Gudjonsson et al^25^ reported a negative association between depression and life satisfaction, they found no significant effects of anxiety and stress on life satisfaction. Given the mixed outcomes of previous studies, the aim of the present study was to investigate the associations among college students’ smartphone use, smartphone addiction, DAS, and SWL to further clarify mental health issues related to excessive smartphone use (Figure 1).

## Materials and Methods

### Study design and setting

Institutional Review Board approval was obtained from the Human Subjects Committee, Human Subjects Office of the sponsoring university, located in the U.S. Midwest. Cross-sectional data were collected by convenience sampling in the fall of 2016 via an online survey of undergraduate students enrolled in one or more courses at the university in the fall of 2016.

### Study participants and size

Undergraduate students were of most interest in this study because they tend to be more intensely involved with smartphones and other technology applications than any other group.^13^ A total of 881 responses to an online survey were obtained (7% response rate). Responses from students with no smartphones (n = 64) and incomplete responses (n = 216) were omitted from the data analysis to prevent biased statistical findings.^26^ The remaining sample size of 601 (n = 601) was included in the analysis, meeting the minimum sample size of 200 for confirmatory factor analysis and structural equation modeling^27^ and also meeting the minimum sample size of 371 as calculated from the population size, with a confidence level of 95% and a margin of error of 5%. The 601 respondents were compared to non-respondents including those who had incomplete responses for a non-response bias check, using school year (i.e., freshman, sophomore, junior, and senior) to match them with focal respondents, which was the only demographic identifier variable in the list obtained from the Freedom of Information Act (FOIA) office.

### Measurement of constructs

*Smartphone use*

This construct was measured via items representing two subdimensions: traditional social communication use (e.g., making/receiving calls, sending/receiving emails, taking/sending photographs) and application-based social network service use (e.g., Twitter, Facebook) derived from Leung.^28^ Respondents were asked how often they utilized their smartphone for these items on a 5-point Likert-type scale (1 = never, 5 = very often). High scores indicated high smartphone use. Cronbach’s alpha coefficients are calculated from the pairwise correlations between items measuring a latent construct and range from 0 to 1 with higher values demonstrating greater internal consistency. Alpha coefficients were used to determine whether a set of scale items meet an acceptable threshold value of reliability (> 0.60).^29^ The alpha coefficients of traditional social communication use and application-based social network service use are 0.61 and 0.65, respectively.

*Smartphone addiction*

Addiction to smartphone use refers to a compulsive reliance on smartphone use to an extent that physiological, mental or emotional outcomes occur. Survey items assessed tolerance and withdrawal symptoms (e.g., “I am always thinking that I have a message on my smartphone”) derived from Cho and Lee.^2^ Respondents expressed how they felt about using their smartphones on a 5-point Likert type scale (1 = strongly disagree, 5 = strongly agree). Higher scores represented high smartphone addiction. The internal consistency reliability is 0.82.

*Depression, anxiety, and stress (DAS)*

This mental health construct represents psychological states and emotions that humans experience in their daily lives. The psychometric properties of various emotions were assessed utilizing the valid and reliable Depression Anxiety Stress Scale (DASS21), with items derived from Antony et al^30^ and Lovibond and Lovibond.^31^ Respondents were asked how often they experienced the mental states described in the items (e.g., “I find it difficult to work up the initiative to do things”) on a 4-point Likert type scale (0 = never, 3 = almost always). Higher scores indicated high emotional distress. The alpha coefficients of depression, anxiety, and stress are 0.90, 0.82, and 0.93, respectively.

*Satisfaction with life (SWL)*

This construct is widely known as an important aspect of subjective well-being.^32^ Items (e.g., “I am satisfied with my life”) derived from the SWL scale^32^ were assessed on a 5-point Likert scale (1 = strongly disagree, 5 = strongly agree). Higher scores indicated high SWL. The internal consistency reliability is 0.87.

### Data analysis

The psychometric properties of the constructs and tests of the proposed model and). hypotheses were evaluated utilizing SPSS 22 and AMOS 22 (IBM Corp., Armonk, New York, USA). The final data (n = 601) with complete responses to individual observed items measuring the hypothesized latent constructs^26^ were included in the initial processes of examining kurtosis and skewness values for the individual observed indicators to determine acceptable normality in the data. Kurtosis values between -3 and +3 are considered acceptable for normality in structural equation modeling.^33^ Also, skewness values between -2 and +2 are considered acceptable for symmetric distributions.^33^ We found that all kurtosis values ranged between -1.60 for “Instagram” and 2.39 for “I feel that life is meaningless” (Table 1) and that all skewness values ranged between -1.57 for “sending/receiving text messages” and 1.74 for “I feel that life is meaningless,” indicating that the individual variables are normally and symmetrically distributed and eligible for data analyses in this study. Thus, the final eligible data (n = 601) were analyzed with a two-stage process; (1) comprehensive measurement model and (2) structural model.^34^

First, confirmatory factor analysis was performed to evaluate a comprehensive full measurement model utilizing pre-specified constructs (i.e., smartphone use, smartphone addiction, DAS, SWL). The presence of an acceptable measurement model fit was assessed using goodness-of-fit indices at a minimum recommended by Kline^27^ and acceptable criteria values, including normed chi-square (*χ*^2^*/df* ≤ 3.0),^27^ root mean square error of approximation (RMSEA) (≤ 0.08),^27,35,36^ standardized root mean square residual (SRMR) (<0.08),^35^ and comparative fit index (CFI, not very sensitive to sample size) (≥ 0.90).^26,27,36,37^ In addition, to determine acceptable internal consistency reliability of the indicators, Cronbach’s alpha coefficient values (≥ 0.60)^29^ for each factor with multiple items were examined. To evaluate adequate convergent validity of the constructs, standardized factor loadings (≥ 0.50) and *t*-values (≥ 1.96)^26,38^ for each item within respective factors were checked. To verify the acceptable discriminant validity of the constructs (< 0.85),^27^ values correlated between the dimensions were checked. If such values are less than .85, the constructs meet the discriminant validity.

Second, structural equation modeling analyses were performed to examine the hypothesized relationships among the latent variables (i.e., smartphone use, smartphone addiction, DAS, SWL). Individual item values of each latent dimension representing the constructs (i.e., smartphone use, smartphone addiction, DAS) were utilized in the structural model. This second-order approach provides information about not only the relative importance of observed items but also the original relationships between the individual items and latent dimensions.^39,40^ The goodness-of-fit indices and criteria values were also evaluated to determine the overall structural model fit.

## Results

### Non-response bias check and participant characteristics

No significant difference (e.g., *χ*² = 3.164, *p* > .05 for school years) between respondents and non-respondents was found, which indicates that the sample likely comprised a representative group of students in the university by year of enrollment. As shown in Table 2, most respondents included in the data analysis were female (n = 374, 63%). The largest group of respondents by year comprised seniors (n = 214, 36%), followed in descending order by juniors (n = 144, 24%), freshmen (n = 135, 22%), and sophomores (n = 108, 18%). Most respondents (n = 399, 67%) were registered for 15 credits or more during the semester of data collection.

### Measurement model testing

Results demonstrated that the comprehensive measurement models fit the data well. As displayed in Table 1, results revealed acceptable fit statistics (*χ*^2^/*df* = 3.451, RMSEA = 0.049, SRMR = 0.047, CFI = 0.913.). Cronbach’s alpha coefficients of the latent constructs ranged between 0.61 and 0.90, meeting an acceptable threshold value of internal consistency reliability (> 0.60).^29^ Convergent validity was met with all items loading on their respective latent factors over .50 except for three items (e.g., .44 for “I am aware of dryness of my mouth”), and all *t*-values were significantly higher than 1.96 (*P* < 0.05).^26,38^ Values correlated between the constructs in Table 3 revealed less than 0.85 except for the value of .86 correlated between anxiety and stress. The correlated constructs met the acceptable discriminant validity value (< 0.85).^27^ These findings indicate that reliable and valid constructs were utilized.

### Structural model testing and structural relationship between constructs

**Figure 2:**
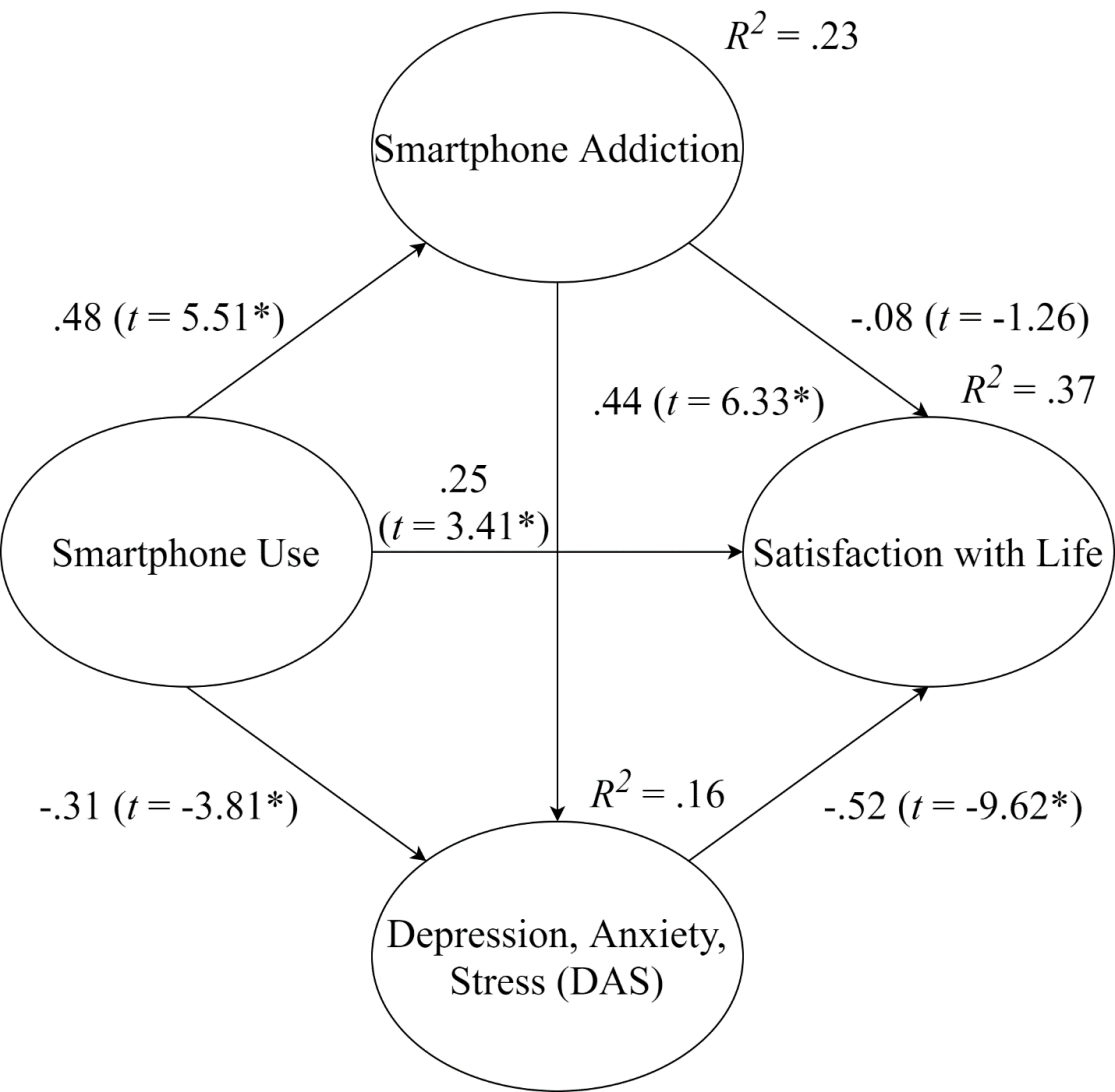
Results of the structural model: tests of hypothesized associations between constructs.
*Note*. Solid lines indicate significant paths (*P < 0.001), Dotted lines indicate non-significant paths* (P >* 0.05), Fit statistics:Chi-square (*χ*^2^)/Degrees of freedom (*df*) = 2.455, Root mean square error of approximation (RMSEA) = 0.049, Standardized root mean squared residual (SRMR) = 0.057, Comparative fit index (CFI) = 0.901

Results of the proposed structural model demonstrated a reasonable model fit (*χ*^2^/*df* = 2.455, RMSEA = 0.049, SRMR = 0.057, CFI = 0.901). Results of the hypothesized relationships are displayed in Figure 2 and Table 4. Results of the test for Hypothesis 1 revealed a significant positive relationship between smartphone use and smartphone addiction (*β* = 0.48, *t* = 5.51, *P* < 0.001). Results of the test for Hypothesis 2 showed a significant negative relationship between smartphone use and DAS (*β* = -0.31, *t* = -3.81, *P* < 0.001). Results of the test for Hypothesis 3 revealed a significant positive relationship between smartphone use and SWL (*β* = 0.25, *t* = 3.41, *P* < 0.001). Results of the test for Hypothesis 4 showed a significant positive association between smartphone addiction and DAS (*β* = 0.44, *t* = 6.33, *P* < 0.001). Results of Hypothesis 5 showed a non-significant relationship between smartphone addiction and SWL (*β* = -0.08, *t* = -1.26, *P* > 0.05). Results of the test for Hypothesis 6 revealed a significant negative relationship between DAS and SWL (*β* = -0.52, *t* = -9.62, *P* < 0.001). Finally, the results revealed that 23% of the variance in smartphone addiction, 16% of the variance in DAS, and 37% of the variance in SWL were explained by their respective preceding construct(s) (see Figure 2).

## Discussion

In this study the structural relationships among smartphone use, smartphone addiction, DAS, and SWL among college students were explored. The results provide evidence that smartphone use is negatively associated with mental health problems, which suggests that it can alleviate such problems. In addition, this study shows that smartphone use has a positive effect on college students’ life satisfaction. However, smartphone addiction, which was positively associated with smartphone use, was found to negatively influence mental health, although it was neither positively nor negatively associated with life satisfaction, as similarly reported by Yang et al.^20^

The findings of this study are aligned with previous studies showing that smartphone use can lead to smartphone addiction, which negatively affects mental health among college students.^3,6,12,13^ For example, De-Sola Gutiérrez et al^41^ conducted a review of the literature on smartphone addiction and found that adolescents and college students who were predisposed to excessive online communication and social networking through smartphones were susceptible to anxiety, irritation, loneliness and stress. The present study extends the body of literature indicating that college students who exhibit smartphone addiction are likely to experience such mental health problems.

On the other hand, in the present study, normal smartphone use was negatively associated with mental health problems, indicating that smartphone use can reduce such health problems. Lepp et al^23^ support this relationship by demonstrating that college students who use smartphones for appropriate amounts of time rather than excessively (e.g., 5 hours a day or frequently to constantly checking them) experience decreased levels of depression, anxiety, and stress. Our interpretation is that appropriate smartphone use may be instrumental in reducing negative psychological problems and concerns. This finding also suggests that the extent to which college students effectively manage their smartphone use can play an important role in whether it influences their mental health in a positive or negative manner. Previous studies have shown links between smartphone addiction and mental health problems (e.g., depression and anxiety) among college students.^16,18^ The results of the present study support this negative relationship between smartphone addiction and mental health among college students.

A few studies have presented mixed or contradictory results in terms of the relationship between smartphone addiction and SWL.^19,20^ The results of the current study support Yang et al’s^20^ finding that smartphone addiction was not related to SWL among college students. However, this study did show that smartphone use was positively related to SWL, indicating that an appropriate amount of smartphone use can help improve college students’ SWL.

Gudjonsson et al^25^ found that anxiety and stress were not significantly associated with life satisfaction among college students. However, Kumar et al^24^ found that emotional distress was negatively related to SWL, which is supported by the findings of this study that college students with high DAS reported diminished life satisfaction. These findings are also consistent with other studies showing that mental problems such as, depression, anxiety, and stress are related to low life satisfaction.^2,10,12^

Some limitations of this study should be noted. First, smartphone use was measured in terms of two subdimensions related to social purposes. There are other purposes for smartphone use such as education, games, and stress reduction, which may be associated with smartphone addiction among college students. Future studies might explore the relationships between a wider range of purposes for using smartphones and smartphone addiction. Second, we acknowledge that because our data on smartphone use and psychological symptoms are based entirely on self-report, there is no objective, clinical assessment of depression, anxiety, or stress, nor an objective measurement of how much college students are actually using their phones. Third, demographic variables such as gender, race, and socio-economic status may have important associations with smartphone use, smartphone addiction, DAS, and SWL among college students, although our preliminary analyses revealed a statistically non-significant relationship between some variables (i.e., gender and smartphone use; school year and smartphone use; gender and SWL). To increase the generalizability of findings, future researchers might further investigate the relationships of demographic variables with smartphone use and health outcomes using data collected from a larger sample of college students. Fourth, smartphone use research with non-student samples is warranted.

## Conclusion

This study is an investigation of how college undergraduates’ smartphone use, smartphone addiction, DAS, and life satisfaction are associated in a structural model. The results revealed significant relationships among these variables. The positive association between smartphone addiction and DAS found in this study emphasizes the importance of the appropriate use of smartphones for the mental health of college students. In addition, this study supports previous findings that appropriate smartphone use can be related to both reduction of DAS as well as improvement of SWL, suggesting the value of effective smartphone use management. Thus, the present study extends the body of literature showing that problematic smartphone use can cause mental health problems and concerns, and that appropriate amounts of smartphone use promote mental health and life satisfaction among college students.

Thus, it is important to engage college students in addressing issues of smartphone addiction. Some research has shown that exercise interventions are efficacious for reducing smartphone addiction, especially among young adults.^11^ Such interventions may be particularly suited for college students, given their access to campus gyms and intramural sports opportunities. It is of equal importance to facilitate health sciences researchers’ and health service providers’ growing awareness of the implications and complications of college students’ uses of smartphones and similar devices such as tablets. Future researchers may consider examining geographic variations (i.e., U.S. south, midwest, northeast, west) in the relationships demonstrated in this study, as well as determining the efficacy of exercise interventions for smartphone addiction in particular geographic regions.

## Acknowledgments

The authors thank the participants of this study who took their time to participate in the survey.

## Funding

No funding was received to support this project.

## Competing interests

The authors declare no potential financial and non-financial relationships and activities in the research, authorship, and publication of this manuscript.

## Ethical approval

We are reporting studies that involved human participants and data. Institutional Review Board approval was obtained from the Human Subjects Committee, Human Subjects Office at Southern Illinois University, Carbondale, Illinois. Data were collected via an online survey of undergraduate students at the university in the fall of 2016. The human Subjects Committee (Dr. Wayne R. Glass, Interim Chair, siuhsc@siu.edu, 618-453-4533) approved the protocol (Protocol Number: 16274).

## Authors’ contributions

NK was involved in the conception and design of the study, including the literature review, data collection, data analysis, and interpretation. NK also wrote most of the manuscript sections. JK helped in the manuscript preparation, critical manuscript review, and manuscript editing. JM wrote some literature sections. JK helped in manuscript evaluation, particularly the discussion section. KK helped evaluate and edit the manuscript.

**Table 1:** Item means, kurtoses, factor loadings (λ), and reliabilities (α) for comprehensive measurement model

**Constructs**	**Mean**	**SD**	**Kurtosis**	**λ**	**t-value**	***P***
*Smartphone Use - Traditional Social Communication Use* ^a^(α = 0.61)	4.03	0.63				
Making/receiving calls	3.82	0.91	-0.68	0.50	-	-
Sending/receiving text messages	4.54	0.72	2.14	0.67	8.32	< 0.001
Taking/sending photographs	3.85	0.99	-0.54	0.59	8.14	< 0.001
*Smartphone Use - Application-Based Social Network Service Use*¹ (α = 0.65)	2.74	1.01				
Twitter	2.18	1.45	-0.70	0.54	-	-
Facebook	3.69	1.30	-0.52	0.53	8.65	< 0.001
Instagram	2.86	1.62	-1.60	0.71	9.66	< 0.001
*Smartphone Addiction - Tolerance and Withdrawal* ^b^(α = 0.82)	2.55	0.75				
The people around me tell me that I use my smartphone too much	1.99	0.95	1.33	0.56	-	-
I feel the urge to use my smartphone again right after I stop using it	2.64	1.19	-1.00	0.78	12.88	<0.001
I have used my smartphone for longer than I had intended	3.56	1.10	-0.21	0.52	9.90	<0.001
I am lacking adequate sleep due to smartphone use at night	2.18	1.10	-0.09	0.61	11.26	<0.001
I am always thinking that I have a message on my smartphone	2.84	1.13	-1.00	0.60	11.14	<0.001
I neglect matters other than smartphone use	2.29	1.00	-0.22	0.62	11.22	<0.001
I can’t stop using my smartphone even when there are many other things to be done	2.34	1.08	-0.32	0.75	12.71	<.001
*DAS - Depression* ^c^(α = 0.90)	0.73	0.65				
I feel that life is meaningless	0.49	0.81	2.39	0.78	-	-
I feel that I have nothing to do look forward to	0.59	0.82	1.17	0.84	22.88	<0.001
I can't seem to experience any positive feeling at all	0.58	0.76	1.34	0.83	22.24	<0.001
I am unable to become enthusiastic about anything	0.74	0.81	0.54	0.72	18.67	<0.001
I feel that I am not worth much as a person	0.77	0.91	0.05	0.80	21.50	<0.001
I feel down-hearted and blue	0.78	0.82	0.53	0.81	21.97	<0.001
I find it difficult to work up the initiative to do things	1.17	0.79	-0.04	0.50	12.47	<0.001
*DAS - Anxiety* ^c^(α = 0.82)	0.70	0.56				
I am aware of the action of my heart in the absence of physical exertion	0.76	0.82	0.09	0.47	-	-
I experience breathing difficulty	0.49	0.74	2.00	0.65	10.31	<0.001
I experience trembling (e.g., in the hands)	0.59	0.80	0.97	0.61	10.00	<0.001
I feel I am close to panic	0.60	0.81	0.86	0.84	11.44	<0.001
I feel scared without any good reason	0.54	0.77	1.25	0.81	11.29	<0.001
I am worried about situations in which I might panic and make a fool of myself	1.31	0.90	-0.67	0.63	10.21	<0.001
I am aware of dryness of my mouth	0.61	0.79	0.81	0.44	8.29	<0.001
*DAS - Stress* ^c^(α = 0.83)	0.95	0.59				
I am intolerant of anything that keeps me from getting on with what I am doing	0.96	0.80	-0.04	0.49	-	-
I feel I am rather touchy	0.76	0.80	0.15	0.59	10.14	<0.001
I find it difficult to relax	1.00	0.89	-0.23	0.73	11.27	<0.001
I find myself getting agitated	0.97	0.78	-0.18	0.72	11.18	<0.001
I feel that I am using a lot of nervous energy	1.05	0.91	-0.59	0.71	11.14	<0.001
I find it hard to wind down	0.84	0.82	0.21	0.67	10.78	<0.001
I tend to over-react to situations	1.10	0.83	-0.11	0.58	10.09	<0.001
*Satisfaction with Life* ^b^(α = .87)	3.32	0.87				
In most ways my life is close to my ideal	3.27	1.00	-0.36	0.79	-	-
The conditions of my life are excellent	3.45	0.98	-0.18	0.75	19.01	<0.001
I am satisfied with my life	3.48	1.02	-0.27	0.88	23.64	<0.001
So far I have gotten the important things I want in life	3.49	1.08	-0.32	0.78	20.52	<0.001
If I could live my life over, I would change almost nothing	2.91	1.22	-1.07	0.64	15.83	<0.001

*Note.* ^a^Measured using a 5-point scale format (1 = Never, 2 = Rarely, 3 = Sometimes, 4 = Often, 5 = Very often).^b^Measured using a 5-point scale format (1 = Strongly Disagree, 2 = Disagree, 3 = Neutral, 4 = Agree, 5 = Strongly Agree). ^c^Measured using a 5-point scale format (0 = Never, 1= Sometimes, 2 = Often, 3 = Almost Always). Measure of model fit: Chi-square (*χ*^2^)/Degrees of freedom (*df*) = 2.307, Root mean square error of approximation (RMSEA) = 0.049, Standardized root mean squared residual (SRMR) = .047, Comparative fit index (CFI) = 0.913.

**Table 2:** Participant characteristics

**Characteristics**	**n**	***%***
Gender		
Female	374	62.8
Male	222	37.2
Student classification		
Freshman	135	22.5
Sophomore	108	18.0
Junior	144	24.0
Senior	214	35.6
Number of credits currently taken		
3-5	3	.5
6-8	5	.9
9-11	16	2.7
12-14	174	29.1
15 or more	399	66.8

**Table 3:** Discriminant validity matrix: values correlated between constructs in comprehensive measurement model

**Constructs**	**TSCU**	**ASNSU**	**TW**	**Depression**	**Anxiety**	**Stress**	**SWL**
TSCU	-						
ASNSU	0.61	**-**					
TW	0.31	0.44	-				
Depression	-0.15	-0.14	0.25	-			
Anxiety	-0.07	-0.02	0.23	0.74	-		
Stress	0.00	-0.02	0.30	0.75	0.86	-	
SWL	0.24	0.19	-0.11	-0.66	-0.44	-0.42	-

*Note.* TSCU = Traditional Social Communication Use, ASNSU = Application-Based Social Network Service Use, TW = Tolerance and Withdrawal, SWL = Satisfaction with Life

**Table 4:** Results of the structural model: tests of hypothesized associations between constructs

**Path**	***β***	***t*** **-value**	***P***
H1: Smartphone Use → Smartphone Addiction	0.48	5.51*	<0.001
H2: Smartphone Use → DAS	-0.31	-3.81*	<0.001
H3: Smartphone Use → Satisfaction with Life	0.25	3.41*	<0.001
H4: Smartphone Addiction → DAS	0.44	6.33*	<0.001
H5: Smartphone Addiction → Satisfaction with Life	-0.08	-1.26	0.209
H6: DAS → Satisfaction with Life	-0.52	-9.62*	<0.001

*Note.* Fit statistics:Chi-square (*χ*^2^)/Degrees of freedom (*df*) = 2.455, Root mean square error of approximation (RMSEA) =0 .049, Standardized root mean squared residual (SRMR) = 0.057, Comparative fit index (CFI) = 0.901. *R*² = 0.23 for Smartphone Addiction, 0.16 for DAS (i.e., Depression, Anxiety and Stress), and 0.37 for Satisfaction with Life. *P<0 .001.
