# Corrigendum to: Examining associations between smartphone use, smartphone addiction, and mental health outcomes: a cross-sectional study of college students Kil N, Kim J, McDaniel JT, Kim J, Kensinger K. Health Promot Perspect. 2021;11(1):36-44. doi: 10.34172/hpp.2021.06

**DOI:** 10.34172/hpp.43496

**Published:** 2024-07-29

**Authors:** Namyun Kil, Junhyoung Kim, Justin T. McDaniel, Jun Kim, Kari Kensinger

**Affiliations:** ^1^Department of Recreation Management and Therapeutic Recreation, University of Wisconsin-La Crosse, La Crosse, WI 54601, USA; ^2^Department of Health & Wellness Design, School of Public Health, Indiana University, Bloomington, IN 47405, USA; ^3^School of Human Sciences, Southern Illinois University, Carbondale, IL 62901, USA; ^4^Therapeutic Recreation of Nebraska, Omaha, NE 68127, USA

 This revises the article “Examining associations between smartphone use, smartphone addiction, and mental health outcomes: A cross-sectional study of college students”.^1^ A correction has been made to Introduction, in the first paragraph. From “the number of college students who use smartphones in the United States (U.S.)” to “the number of people who use smartphones in the United States (U.S.)”.

 The authors apologize for this error and state that this does not change the scientific conclusions of the article in any way. The first published version of the article has been updated.
